# Deep Learning-Based Approach for Automatic Defect Detection in Complex Structures Using PAUT Data

**DOI:** 10.3390/s25196128

**Published:** 2025-10-03

**Authors:** Kseniia Barshok, Jung-In Choi, Jaesun Lee

**Affiliations:** 1 Research Institute of DNA+, Changwon National University, Changwon 51140, Republic of Korea; kseniia@changwon.ac.kr; 2 School of Sarim Honors, Changwon National University, Changwon 51140, Republic of Korea; 3 School of Mechanical Engineering, Changwon National University, Changwon 51140, Republic of Korea

**Keywords:** phased array ultrasonic testing (PAUT), defect detection, deep learning, CATT-S

## Abstract

This paper presents a comprehensive study on automated defect detection in complex structures using phased array ultrasonic testing data, focusing on both traditional signal processing and advanced deep learning methods. As a non-AI baseline, the well-known signal-to-noise ratio algorithm was improved by introducing automatic depth gate calculation using derivative analysis and eliminated the need for manual parameter tuning. Even though this method demonstrates robust flaw indication, it faces difficulties for automatic defect detection in highly noisy data or in cases with large pore zones. Considering this, multiple DL architectures—including fully connected networks, convolutional neural networks, and a novel Convolutional Attention Temporal Transformer for Sequences—are developed and trained on diverse datasets comprising simulated CIVA data and real-world data files from welded and composite specimens. Experimental results show that while the FCN architecture is limited in its ability to model dependencies, the CNN achieves a strong performance with a test accuracy of 94.9%, effectively capturing local features from PAUT signals. The CATT-S model, which integrates a convolutional feature extractor with a self-attention mechanism, consistently outperforms the other baselines by effectively modeling both fine-grained signal morphology and long-range inter-beam dependencies. Achieving a remarkable accuracy of 99.4% and a strong F1-score of 0.905 on experimental data, this integrated approach demonstrates significant practical potential for improving the reliability and efficiency of NDT in complex, heterogeneous materials.

## 1. Introduction

Structural integrity of materials and components is a critical concern across industries such as aerospace, automotive, energy, marine, and construction. In this study, both welded specimens and composite materials are considered as representative cases for evaluating automatic defect detection approaches, since each presents distinct challenges, including high sensitivity to internal defects. In welds, flaws such as cracks, porosity, inclusions, or lack of fusion may appear due to improper technique, material impurities, or environmental conditions during fabrication [1,2]. In composites, the heterogeneous and anisotropic structure makes them vulnerable to delaminations, voids, and fiber misalignments that may not be visible on the surface [3,4,5,6,7,8,9,10,11], while large external defects are relatively easy to identify, small or hidden flaws are far more dangerous, as they may propagate over time and cause catastrophic failures. Early detection of such defects enables timely repair or replacement, extends component service life, reduces costs, and contributes to environmental sustainability by supporting remanufacturing [12].

Traditional destructive methods for evaluating mechanical strength are unsuitable when every manufactured part must be tested, which is why nondestructive testing (NDT) is a fundamental approach for quality assurance [13]. Various NDT techniques exist, including visual inspection, dye penetrant testing, magnetic particle testing, eddy current testing, radiography, and acoustic emission testing [13,14,15]. Radiography, while highly sensitive, introduces radiation hazards, whereas ultrasonic testing (UT) offers a safe and effective alternative [15]. UT employs high-frequency acoustic waves to detect internal discontinuities and has gained wide industrial use due to its accuracy and applicability even when only one-sided access is possible [16].

Phased array ultrasonic testing (PAUT) represents a significant advancement over conventional UT by using electronically steerable transducer arrays that enable beam steering and focusing over a wide angular range [3,4,17,18]. Phased Array Ultrasonic Testing (PAUT) is based on the timing (phase) of ultrasonic transducer pulses, which can be electronically steered, focused, and scanned without mechanical probe movement. This improves sensitivity, resolution, and coverage, especially for geometrically complex welds or anisotropic composite materials [5].

To validate inspection strategies and to prove reliability of use, simulation of PAUT to improve understanding of ultrasonic beam behavior studies with a focus on comparisons between PAUT and simulation have been conducted on irregular structure welds [19], dissimilar weld joints [20,21], and thin-walled pipes [22], confirming the possibility of using CIVA as an additional tool for planning and verifying PAUT inspection.

PAUT data can be displayed as A-scans, B-scans, C-scans, or S-scans, providing rich information about material structure and potential defects. However, this richness comes at the cost of significantly increased data complexity. Interpretation of PAUT scans requires expertise and remains time-consuming, even for specialists [6]. For composite structures, additional challenges arise due to heterogeneity and anisotropy, making it difficult to separate genuine defects from benign structural reflections [3,4,5,6,7,8,9,10,11]. This complexity makes manual interpretation even more challenging and underscores the urgent need for automated solutions.

In response to these challenges, a growing body of research has focused on machine learning (ML) and deep learning (DL) for automated flaw detection; while early approaches demonstrated promising results, they often faced challenges with limited generalizability when applied to real-world data collected with different equipment or under different conditions. Later work expanded to multiple defect classes and data formats, such as OPD files, achieving high accuracy in classification tasks.

Advanced PAUT processing methods like Sampling Phased Array (SPA) and Total Focusing Method (TFM) improve spatial resolution and imaging fidelity, but still rely on manual interpretation and expert judgment, leaving throughput and reproducibility constraints in place [7,8,9,10]. Signal-processing approaches. Wavelet-based features with Shannon entropy and model-based entropy methods have shown promise for noisy ultrasonic/PAUT signals and in situ indication, but typically require parameter tuning and human confirmation, limiting scalability [23,24,25]. Signal-processing approaches. Wavelet-based features with Shannon entropy and model-based entropy methods have shown promise for noisy ultrasonic/PAUT signals and in situ indication, but typically require parameter tuning and human confirmation, limiting scalability [23,24,25]. Similarly, in the broader field of NDT image processing, filtering algorithms such as the median filter have been widely applied to enhance defect clarity in radiographic images [26].

Supervised deep learning for PAUT. Early ML/DL studies on welds demonstrated accurate classification for specific defect types, yet often struggled to generalize across scanners, setups, or materials. Subsequent works expanded to multiple defect classes and OPD formats, achieving strong accuracy but retaining sensitivity to domain shifts [23,24,25,27,28,29,30,31]. Unsupervised and multimodal directions. Recent unsupervised learning for composite PAUT and multimodal fusion with thermography indicate benefits for heterogeneous materials, but add integration complexity and do not remove the need for robust sequence-level modeling of inter-beam context [32,33,34].

Despite these advancements, most existing models have struggled to explicitly model the interdependencies across a sequence of multiple A-scan signals, a critical aspect for accurate defect characterization in complex materials.

FCNs are capacity-limited for structured signals. CNNs capture local morphology but have fixed receptive fields and weak long-range dependency modeling. Attention-based multi-signal classifiers improve inter-signal reasoning but still need designs that balance localized feature extraction with global context [35,36,37]. Medak et al. [38] addressed context by merging features from neighboring B-scans (Conv2D/ConvLSTM fusion). They reported only modest mAP gains and noticeably slower inference, since sequence fusion adds compute on top of a 2D detector. Transformers have also been explored for ultrasonic signal processing, but mainly for tasks like deconvolution rather than PAUT sequence classification [39]. Most recent PAUT DL works focus on B/C-scan image detectors or single A-scan classification [34,40,41,42,43]; they do not explicitly model inter-beam dependencies across a sequence of A-scans. In our work, we target these gaps: we make SNR analysis automatic (gates from derivatives, thresholds from σ) to reduce operator dependence, and we introduce CATT-S, where a CNN extracts per-signal features and a Transformer with self-attention models dependencies across the full A-scan sequence.

To address these limitations and improve prediction accuracy under varying noise and complex geometries, we propose the CATT-S model, a sequence model that jointly processes multiple A-scan signals to capture inter-beam context. Unlike prior studies, this paper introduces a parameter-free SNR baseline and a sequence model (CATT-S), and provides a head-to-head comparison against FCN/CNN on both simulated welds and large-scale experimental OPD composites.

The main contributions of this work are as follows:

1. The development and validation of an automated, parameter-free SNR algorithm for rapid defect indication.
2. A comparative analysis of three distinct deep learning architectures (FCN, CNN, and CATT-S) on a diverse PAUT dataset.
3. The proposal of a novel CATT-S model that effectively combines local feature extraction with global context modeling through self-attention, achieving state-of-the-art performance in complex PAUT data analysis.
4. The use of a combined simulated and large-scale experimental dataset to demonstrate the practical viability and generalizability of the proposed methods.

## 2. Materials and Methods

### 2.1. Dataset Collection and Processing

Two types of datasets were used to evaluate the proposed approaches. For welded structures, both simulated and experimental data were collected. The simulated dataset was generated using CIVA software (2023SP4 version), which provided controlled conditions for creating weld specimens with artificial flaws and noise levels [29]. Artificial defects, including cracks, porosity, and inclusions, were primarily placed in critical regions such as the weld toe and weld root to reflect realistic conditions. In total, approximately 200 simulated S-scan samples were collected, each consisting of 39 A-scan signals, and subsequently augmented using signal recombination and noise map variations to expand the dataset by a factor of 2.5. Experimental welded-plate data were obtained using phased array ultrasonic testing (PAUT) equipment (OmniScan ™ X3, Olympus, Quebec, QC, Canada), with signals stored in the OPD format [30].

For composite materials, real-world datasets were collected from carbon fiber reinforced polymer (CFRP) and other composite specimens. These datasets included multiple types of structural defects, such as delaminations, inclusions, and porosity, and were acquired under industrial inspection conditions [31]. To manage and analyze these varied data types efficiently, a dedicated software tool was developed. The composite datasets posed additional challenges due to the anisotropy and heterogeneity of materials, leading to higher structural noise compared to metallic welds.

**Figure 1:**
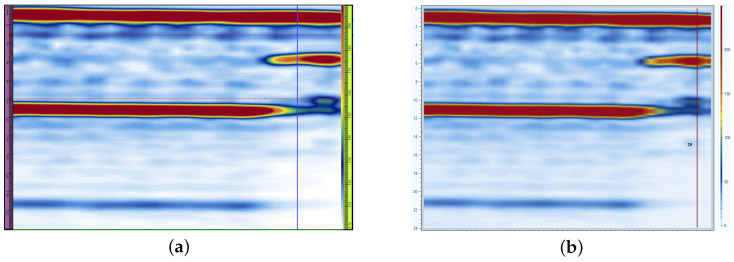
Comparison of S-scan for 199 mm scan step of same testing sample in OmniPC software (OmniPC™ 6.0.0) (**a**) and using developed software sw (**b**).

A dedicated software tool was developed to handle PAUT data in OPD format. This tool served as a critical component of the overall pipeline, enabling the seamless integration of diverse datasets into the proposed analysis methods. Its development ensured that both simulated and real-world experimental data could be consistently processed and utilized for training and evaluation. As one of the most popular formats for PAUT data, Omnipage documents (OPD) were specifically targeted for this purpose. One of the most popular formats to store PAUT data is Omnipage documents (OPD) files collected using OmniScan equipment (OmniScan ™ X3, Olympus, Quebec, QC, Canada) and FPD files collected using FocusPX equipment (FocusPX, Olympus, Quebec, QC, Canada). So, for the first step of PAUT data analysis, experimental data files should be correctly read, and all indications with possible defects should be marked. For this purpose, an algorithm to read OPD data files was developed. This algorithm reads OPD and FPD as binary files and stores all information (sound velocity, angle range, time range, amplitude, etc.). Based on collected information, it is possible to create S-scan and A-scan images. To prove the correctness of the algorithm for reading data from OPD files, a comparison was done between the OmniPC and the newly developed algorithm, using S-scan of the same data file for the same scan position Figure 1.

**Figure 2:**
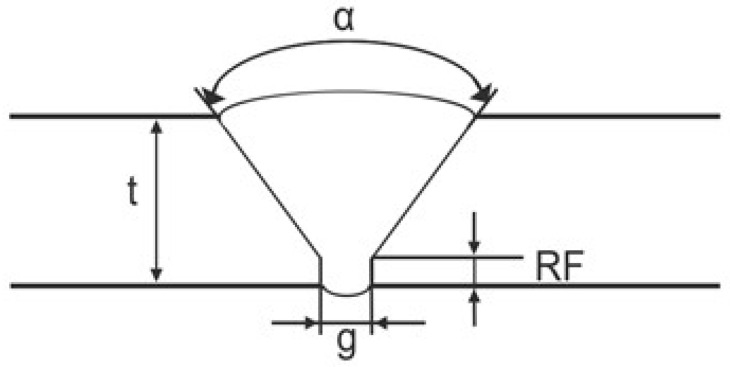
Schematic weld profile with variable parameters.

To develop a deep learning-based approach for automatic defect defection, datasets of welded and composite structures were prepared. We first needed to develop an approach for automatic defect detection that would work with one type of weld shape before expanding it to apply to other types of weld shapes. Due to the limited amount of experimental data available, we decided to expand the dataset using CIVA simulation. Thus, for CIVA modeling, we focused on butt joint weld shape such that the weld profiles match those used to collect experimental PAUT. Simulation included variation in the main parameters: root face RF (0–3 mm), gap ζ (1–4 mm), thickness *t* (5.6–18 mm), and angles α (70° and 75°) (Table 1). Due to the artificial nature of the generated data, only 3 types were selected for defect simulation, such as cracks, porosity, and inclusions which were placed in typical regions like weld root and toe. Incomplete penetration (IP), lack of fusion (LF), and undercut (UC) defects were hard to simulate in the CIVA version we had, so we focused on those defects that could be reliably generated to expand our dataset. Non-defective welds with varied geometrical profiles were also included to ensure the dataset captured both flawed and healthy indications. Structural noise was also incorporated with different amplitudes and densities to approximate real inspection conditions. In total, approximately 200 simulated S-scan samples were collected, each consisting of 39 A-scan signals, and subsequently augmented using signal recombination and noise map variations to expand the dataset by a factor of 2.5, preserving variability and complexity for neural network training. A schematic of the weld profile with variable parameters is shown in Figure 2.

For further use, collected simulated and experimental datasets were separately split into train, validation and test subsets, and then each subset of simulated data was mixed with the corresponding subset of experimental data, forming final subsets. This prevents data leakage between simulated and real weld samples. For composites, only experimental data in OPD and FPD format were collected from CFRP specimens. Due to customer restrictions, we cannot disclose detailed material properties such as carbon content or manufacturer. Data files were processed, each containing approximately 18,000 A-scan signals, resulting in more than 50,000 training samples and about 10,000 test samples after preprocessing and labeling.

By using simulated weld datasets with large real-world composite datasets, the study established a broad foundation for evaluating both non-AI and DL-based detection approaches on PAUT data collected from structurally diverse materials. Because experimental data were received in OPD and FPD format, a special tool was developed in our software to process these files. This tool allows data preprocessing and saving them to JSON format. Thus, multiple JSON files were generated with preprocessed signals (normalized to the same signal length and marked where defects are and where they are not). Then these JSON files were split into training and testing subsets, so signals from one file were not mixed across subsets, preventing leakage.

With these datasets prepared, the next step was to apply and evaluate different defect detection methods, beginning with the enhanced signal-to-noise ratio (SNR) approach as a non-AI baseline before advancing to deep learning models.

### 2.2. Enhanced SNR-Based Method

This section describes an enhanced signal-to-noise ratio (SNR)-based method implemented in parallel with the deep learning (DL) models. This approach differs from conventional SNR analysis, which relies on manual depth gating, by automatically determining the depth gates. This is achieved through the following three-step process:

1. Constructing depth scans (D-scans), which involves applying max-projection across the beams.
2. Collapsing the D-scan into a single depth-accumulated signal by averaging along the scanning axis.
3. Performing derivative analysis to automatically locate acoustic interfaces.

This process allows for automatic gating and flaw indication directly from PAUT data without requiring manual parameter tuning; while effective in many cases, the method is not universally applicable and may be less reliable for data with excessive noise or for defects like large pore zones.

### 2.3. Deep Learning Models

Three neural network architectures were investigated for their ability to classify and localize defects. A Fully Connected Network (FCN) for direct signal classification, a convolutional neural network (CNN) for local feature extraction from A-Scan and S-Scan inputs, and a convolutional neural network with Attention Temporal Transformer for (CATT-S), which first extracts local features with a convolutional block and then applies Transformer self-attention to jointly model inter-beam dependencies across multiple A-scans. The FCN was utilized as a simple baseline model to establish foundational performance and confirm the feasibility of a deep learning approach for this task. The CNN was then employed to exploit its strength in automatically extracting salient, local features from the signal, which is crucial for distinguishing defect patterns. Finally, in CATT-S, a convolutional feature extractor first processes each A-Scan to produce localized embeddings, and a Transformer encoder with self-attention then jointly models the sequence of A-Scans to learn inter-signal dependencies and global context across signals. This design captures complex inter-signal relationships and subtle defect features, which is essential for overcoming the limitations of models with a fixed receptive field, while retaining the strengths of convolution for local pattern extraction. All models were trained using both simulated and real OPD datasets, with augmentation applied to improve generalization. Training and validation were carried out on separate subsets to prevent overfitting.

## 3. SNR-Based Automatic Defect Detection

### 3.1. Automatic Depth-Gate Detection

As a non-AI baseline, an enhanced signal-to-noise ratio (SNR)-based method was implemented to provide automatic defect detection in PAUT data. Unlike conventional SNR evaluation, which relies on operator-defined gates and manual parameter tuning, the developed approach introduces an automatic procedure for depth-gate detection and statistical defect characterization. Original phased-array acquisition delivers the following three-dimensional matrix:

(1) $$S=\left\{S\left(x,z,n\right)\right\}\;\left\{\begin{array}{c} x=1,\ldots ,M\;(\mathrm{scan}\;\mathrm{index})\\ z=0,\ldots ,Z_{\max}-1\;(\mathrm{time-of-flight}\;\mathrm{index})\\ n=1,\ldots ,N\;(\mathrm{beam}\;\mathrm{index}) \end{array}\right.$$

For each (x,z), keep only the maximum echo over all beams,

(2) $$D\left(x,z\right)=\max_{1\leq n\leq N}S\left(x,z,n\right),$$

to obtain a D-scan where $D\in R^{M\times Z_{\max}}$. The max-projection preserves specular reflections that might occur in only a few beams while suppressing incoherent noise [44]. To characterize the specimen globally, the D-scan is collapsed along the scan axis by averaging

(3) $$g\left(z\right)=\frac{1}{M}\sum_{x=1}^{M}D\left(x,z\right).$$

The resulting one-dimensional depth profile g(z) has the same length as a single A-scan and represents the mean energy returned from each depth. Derivative analysis is then used to highlight acoustic interfaces. The first ∇g(z) and second Δg(z) discrete derivatives are computed as in Equations (4) and (5) [44].

(4) $$\nabla g\left(z\right)=\frac{dg}{dz}\approx g\left(z+1\right)-g\left(z-1\right).$$

(5) $$\Delta g\left(z\right)=\frac{d^{2}g}{dz^{2}}\approx g\left(z+1\right)-2g\left(z\right)+g\left(z-1\right).$$

Computing the first derivative ∇g(z) converts each step into a pair of extreme: a large positive lobe at the rising edge and a negative lobe at the trailing edge. These extremes quantify the magnitude of the slope and hence localize the regions where the acoustic impedance changes most rapidly. While in the second derivative Δg(z), each steep rise produces one narrow positive curvature peak centered on the interface. To discard the noise peaks, a 25% threshold is defined, and the remaining peaks are used for gate setup.

(6) $$T=\frac{1}{4}\Delta g\left(z\right).$$

**Figure 3:**
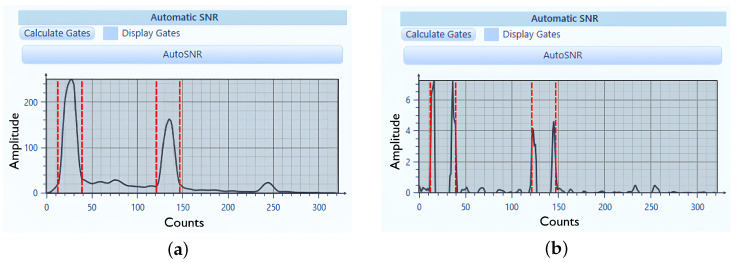
Original signal accumulated from D-scan (**a**) and calculated second order derivative signal (**b**).

The remaining peaks define depth gates, expressed as

(7) $$P={\left\{\left[z_{2i},z_{2i+1}\right]\right\}}_{i=0}^{L-1},$$

where each interval covers the positive curvature zone of one reflector Figure 3. In Figure 3 dashed lines show the start and end of the first (top) and second (bottom) reflection peacks. So, to display the inner structure of material, the gates for C-scan plot are set to be between 2nd and 3rd dashed line: end of top reflection and start of bottom reflection of composite PAUT.

### 3.2. Signal-to-Noise–Based Defect Characterization

Once the gates are defined, SNR analysis is performed on the ultrasonic C-scan. Local amplitudes are normalized by the maximum admissible amplitude $A_{\max}$,

(8) $$p\left(i,j\right)=\frac{D(i,j)}{A_{\max}}\times 100,$$

where D(i,j) is the measured amplitude at spatial coordinates (i,j). Within a selected region Ω, the first and second statistical moments (Equations (9) and (10)) are computed as follows:

(9) $$\mu =\frac{1}{N}\sum_{(i,j)\in \Omega }p\left(i,j\right),$$

(10) $$\sigma =\sqrt{\frac{1}{N}\sum_{(i,j)\in \Omega }{\left(p(i,j)-\mu \right)}^{2}},$$

where *N* is the number of pixels in Ω. Defect thresholds are then defined as a confidence interval, as commonly used in ultrasonic testing [44],

(11) $$S_{\min}=\mu -k\sigma ,\;S_{\max}=\mu +k\sigma ,$$

with *k* set adaptively to σ in the automatic mode. For automatic SNR analysis, we set the value of parameter k adaptively to σ. This choice was made after testing on real PAUT data with different noise levels, specimen geometries and inspection conditions. In most cases using σ as a scaling factor adapts thresholds to the noise spreads, providing sufficient defect markup. If small false-positive defections appear (due to noise variations), they can be removed later from the report table (in developed software) by setting up a threshold for minimal defect size. Pixels above $S_{\max}$ are flagged as high-contrast defects, while pixels below $S_{\min}$ correspond to low-amplitude anomalies. The defective areas are then estimated as

(12) $$A_{<}=A_{\mathrm{tot}}\frac{n_{<}}{N},\;A_{>}=A_{\mathrm{tot}}\frac{n_{>}}{N},$$

where $n_{<}$ and $n_{>}$ are the counts of pixels below $S_{\min}$ and above $S_{\max}$, and $A_{\mathrm{tot}}$ is the total physical area of the inspected region.

**Figure 4:**
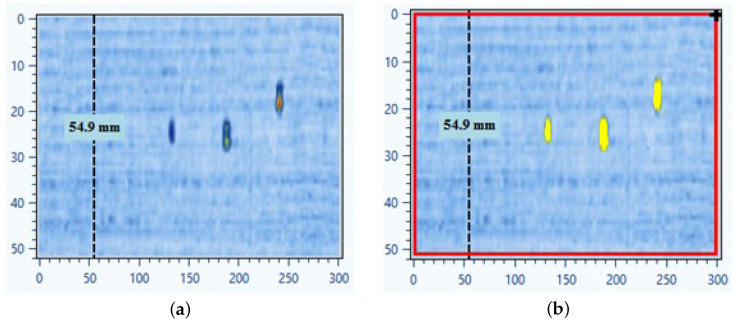
Original C-scan (**a**) and calculated defective area by automatic SNR (**b**).

This procedure enables robust automatic defect zone delineation by combining derivative-based depth gating with statistical amplitude analysis. The method effectively reduces reliance on manual parameter tuning, making it more practical for large-scale inspections. The main improvement of our SNR-based method is that it was made automatic and parameter-free. In standard SNR analysis, the operator usually sets analysis gates for proper C-scan plot view and thresholds manually. In our version gates are calculated automatically from the signal derivative profile, thresholds are set adaptively using k = σ, and amplitudes are normalized. Also, in software small false-positive detections can be removed later via setup of a minimal defect size threshold. Applied to simulated weld datasets, the algorithm successfully detected defective zones in C-scans within automatically calculated gates. When tested on composite OPD datasets, it also demonstrated the ability to highlight defect-like zones in anisotropic CFRP Figure 4. In Figure 4, we presented an example of defective zone detection using an improved approach with automatic gates and SNR parameters setup—C-scan plotted after setting automatically calculated gates (a) and after applying SNR with automatically tuned parameters with defective zones marked yellow (b).

**Figure 5:**
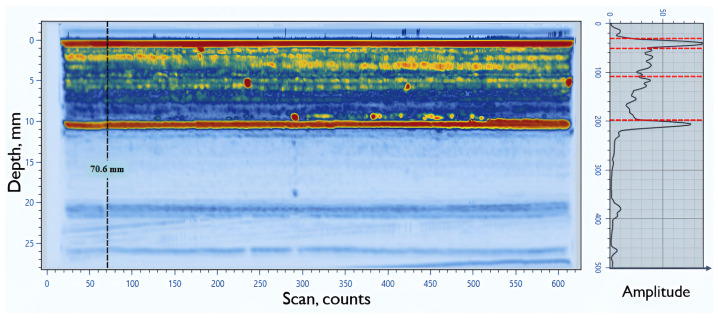
D-scan of highly noised data with wrong automatic gates calculation.

But in data with very high noise levels or large pore zones, automatically calculated gates are usually wrong (Figure 5), so they should be manually adjusted before applying SNR analysis. In pore zones reflections are spread, and amplitude can be close to noise, so thresholds based on k ≈ σ do not always isolate them. In very noisy data σ becomes large and thresholds expand, which reduces sensitivity to real defects and can mark structural noise as defects.

While computationally efficient and interpretable, the SNR-based method is not universally applicable. It serves as a fast baseline, but more advanced deep learning approaches are required to achieve reliable classification and localization across diverse materials and geometries.

## 4. Deep Learning Architectures for Defect Detection

### 4.1. Fully Connected Neural Network

**Figure 6:**
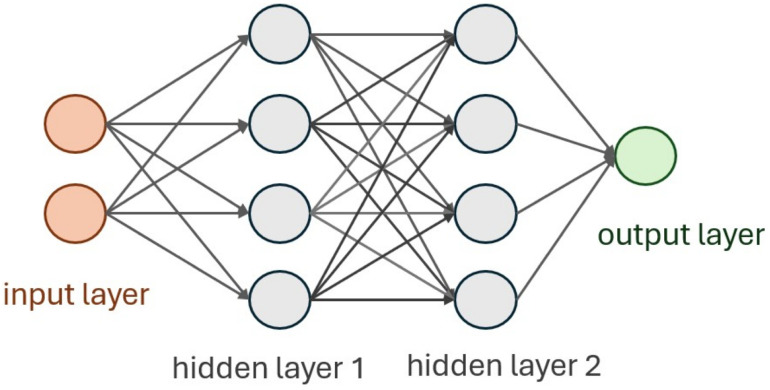
Schematic representation of the common structure of FC NN.

One of the most popular uses of NN models for classification tasks in the case of working with 1D signals is a fully connected neural network (FC NN). The core component is Dense layers, where each neuron from one layer is connected to each neuron of the previous and following layer Figure 6.

**Figure 7:**
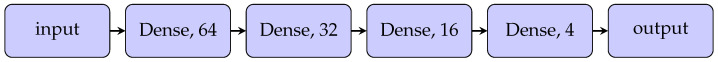
Schematic representation of FC NN designed for defect detection algorithm.

For the prepared dataset, an FC NN structure with four hidden Dense layers was chosen Figure 7. After each hidden layer except the first, the rectified linear unit (ReLU) activation function was used. The output layer contains one neuron with a sigmoid activation function, as the main goal is binary classification of defect and non-defect signals. The Adam optimizer with 0.0001 learning and the binary cross-entropy loss function [45] give better results for the chosen architecture of FC NN.

**Figure 8:**
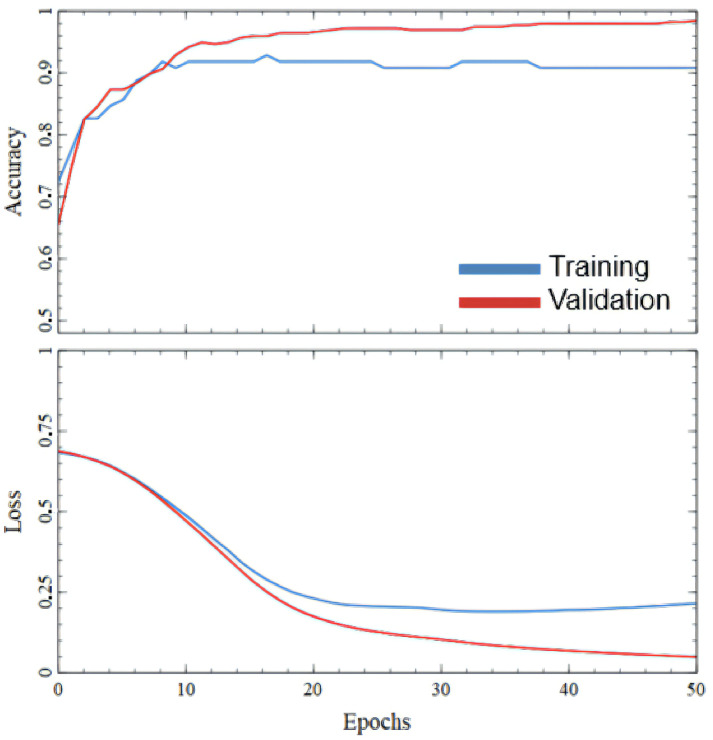
Plot of training and validation accuracy and losses changing in FC NN training process.

FC NN was trained for 50 epochs with batch size 16. The maximum validation accuracy was 90.82%, whereas training accuracy reached 80.8% Figure 8. Increasing the number of epochs led to higher training loss, indicating overfitting and making NN predictions unreliable. Such low testing accuracy is obviously not enough for practical use in real-world cases. More fundamentally, because an FC NN processes each input as a flat vector, it lacks inductive bias for spatial or temporal structure and therefore does not encode neighborhood or sequence information across beams or along depth. These limitations motivate the use of a CNN to capture local morphology and CATT-S to jointly model inter-beam dependencies via self-attention.

### 4.2. Convolutional 1D Neural Network

**Figure 9:**
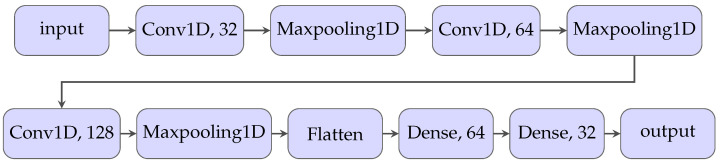
Schematic representation of CNN designed for defect detection algorithm.

The next attempt was to use Convolutional 1D Neural Networks (CNN). The core component is the convolution layer, which extracts salient features from the signal that are essential for differentiating between defect and non-defect signals. The prepared dataset does not contain too many samples, so the final CNN should not consist of too many layers to prevent overfitting. The final CNN contains three Convolutional 1D layers with a MaxPooling 1D layer after each convolution Figure 9.

**Figure 10:**
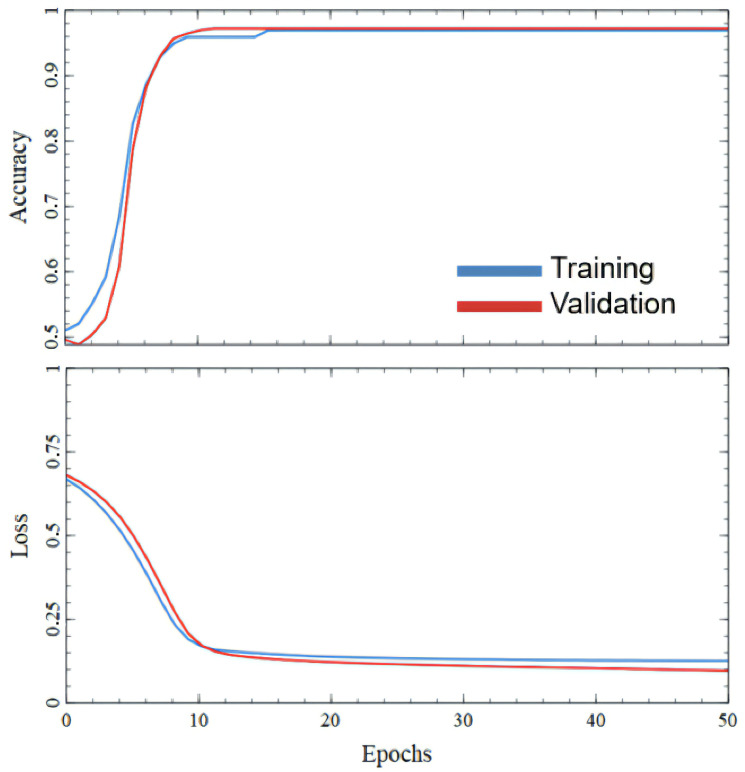
Plot of training and validation accuracy and losses changing in CNN training process.

Like FC NN, the output layer is also represented by a single neuron with a sigmoid activation function. The ReLU activation function was used after each Convolution 1D and Dense layer. The Adam optimizer with a learning rate of 0.0001 was used in the training process with binary cross-entropy as the loss function. The training process took 50 epochs with 16 batch sizes. The maximum achieved validation accuracy is 97.94%, while testing accuracy is 94.9% Figure 10. This is the promising result for practical application of the defect detection algorithm based on this CNN.

Experiments confirmed that the CNN delivered higher accuracy than the FC NN, particularly on noisy weld data. However, when applied to a single A-scan, a 1-D CNN has a fixed receptive field along depth and does not model cross-beam context. In PAUT, echoes from the same reflector appear across sequences of beams with varying arrival times and amplitudes; these patterns are non-stationary and depend on geometry and probe steering. For our welded dataset, this per-A-scan modeling was often sufficient, but for composite datasets, with stronger structural noise and anisotropy, capturing inter-signal relationships is essential. which requires processing a sequence of A-scans jointly, rather than treating each scan independently.

### 4.3. Transformer-Based Neural Network

**Figure 11:**
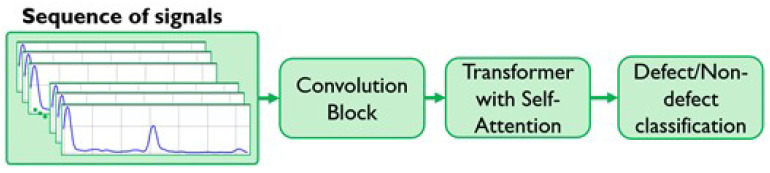
Schematic simplified architecture of CATT-S model.

To overcome the inherent limitations of CNNs, particularly their fixed receptive field and inability to model long-range dependencies, we propose a novel hybrid architecture: the Convolutional Attention Temporal Transformer for Sequences (CATT-S). This model is specifically designed to leverage the strengths of both convolutional feature extraction and the self-attention mechanism of a Transformer Figure 11. The architecture consists of a convolutional block followed by a Transformer encoder. The convolutional layers first process each A-scan signal in parallel, acting as a robust feature extractor to capture local morphology within the PAUT signal. These features are then fed into the Transformer encoder.

The key improvement lies in the self-attention mechanism of the Transformer. Unlike CNNs, which only process features within a limited, local window, the self-attention mechanism computes the relationship between every pair of A-scan signals within a given sequence. This enables the model to effectively capture long-range inter-beam dependencies and global context across the entire PAUT scan. For example, the model can learn to correlate a subtle feature in one beam with a much stronger reflection in a distant beam, allowing it to differentiate a genuine defect from benign structural noise or material anisotropy. This is particularly critical for analyzing complex composite materials, where a holistic understanding of the entire signal sequence is necessary to accurately identify defects.

In developed CATT-S, the CNN block extracts features for each signal in the input sequence separately. The length of each signal in the sequence is always constant—it is necessary for proper feature extraction by CNN. For each signal, one feature vector $f_{i}$ is produced, and for the whole sequence, we obtain *F* = [$f_{1},f_{2},\ldots ,f_{T}$]. Then in the Transformer self-attention block, linear projections are computed as follows: $Q=FW_{q},K=FW_{k},V=FW_{v}$. Projection weights ($W_{q},W_{k},W_{v}$) are constant, but resulting projections depend on sequence size *T*. The attention matrix *A* is then calculated as shown in Equation (13) [46] and has size T×T.

(13) $$A=\mathrm{softmax}\;\left(\frac{QK^{T}}{\sqrt{d_{k}}}\right).$$

Each value in this matrix shows the relation between one signal and another in the sequence. These weights alone represent normalized probabilities, but final context-aware features are produced only after multiplication by value matrix V. This operation makes each output vector a weighted combination of all input features, where weights are controlled by attention scores. In this way attention can build a context window that is not fixed like in CNN, but adaptive to all relations across the sequence. When a defect echo appears in several neighboring signals, their attention weights are reinforced, while isolated local anomalies not repeated in neighbors get counted as noise. CATT-S was trained for 15 epochs with batch size 8, sequence length 50, and the AdamW optimizer ($\mathrm{lr}=8\times 10^{-4}$, weight decay =0.015) with BCE loss in Equation (14) [45].

(14) $$L_{\mathrm{BCE}}\;=\;-\;\frac{1}{N}\sum_{i=1}^{N}[\;y_{i}\log\left(p_{i}\right)+\left(1-y_{i}\right)\log\;\left(1-p_{i}\right)\;],$$

where $p_{i}\in \left(0,1\right)$ is the predicted probability and $y_{i}\in \left\{0,1\right\}$ is the ground-truth label. During the training stage we experimented with different sequence sizes (from 30 to 100), while signal length was always frozen to 320, which is necessary for CNN feature extraction. Considering specifics of experimental PAUT data, we decided to fix sequence length to 50 as an optimal balance. Technically the model can accept variable sequence lengths as long as the NN graph is not frozen, because the projection weights remain the same and only the matrix sizes change. However, for practical deployment, we had to convert the trained NN into ONNX format for integration into developed software. In this stage, the NN graph freezes, and the input sequence length must remain constant (50). To improve training stability on a relatively small dataset, we applied dropout regularization, multi-head attention, early stopping, and moderate hidden layer sizes. Also, overlapping signal sequences were generated using a sliding window to augment the dataset and increase sample variability.

**Figure 12:**
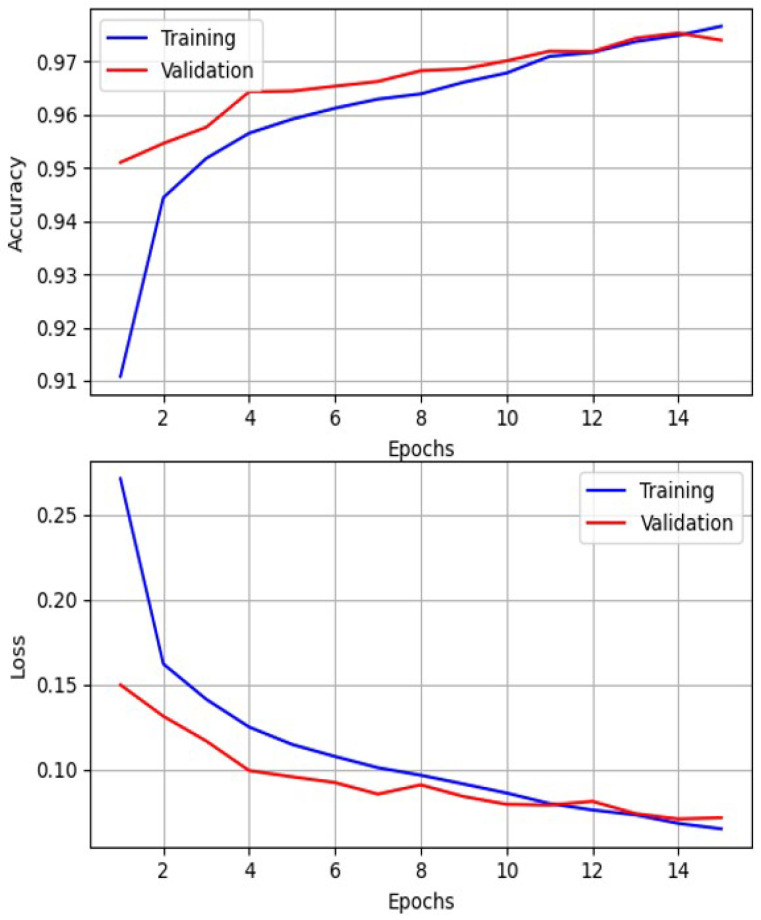
Plot of training and validation accuracy and losses changing in CATT-S training process.

As shown in Figure 12, both training and validation loss decreased steadily while accuracy increased, reaching a validation accuracy of about 97.5%. The training and validation curves remained close throughout, indicating stable convergence and good generalization without signs of overfitting. These results confirm that the convolutional feature extractor combined with self-attention effectively captures both local and long-range features in PAUT sequences.

**Figure 13:**
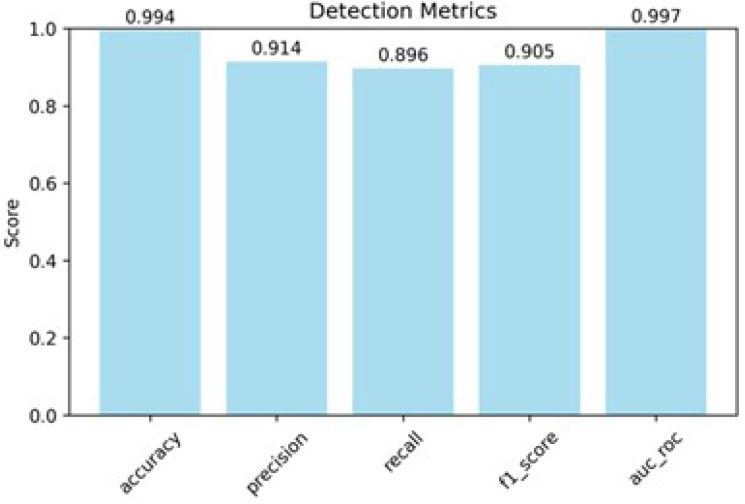
Evaluation metrics of CATT-S on experimental PAUT data.

On experimental PAUT data of complex structures, CATT-S achieved 99.4% accuracy (Figure 13), indicating that almost all samples were classified correctly. On experimental PAUT data of complex structures, among all CATT-S predictions labeled as defects, 91.4% were true defects (precision) with a recall of 0.896—most true defects across all are found, giving an F1-score of 0.905. The ROC–AUC of 0.997 indicates near-complete separability between defect and non-defect signals across thresholds, enabling operating points that keep both false positives and missed defects low.

## 5. Conclusions

This study describes a complete pipeline for automatic defect detection in PAUT data. Due to the main focus of the paper being to improve the process of defect detection, PAUT of butt joint welds was chosen due to its prevalence, and PAUT of composite materials was chosen. First, the well-known SNR method was improved to become automatic and parameter-free. For proper C-scan plot view depth gates are calculated automatically from the signal derivative profile accumulated from D-scan, thresholds are set adaptively, and amplitudes are normalized; while improved SNR algorithm can be applied for successful automatic defect detection, it still has difficulties working with highly noisy data or PAUT of specimens with large pore zones. Thus, as the next step, a DL-based approach was developed. FCN confirmed feasibility but lacked capacity, and CNN achieved strong accuracy (about 95%) but was limited by its fixed receptive field. The proposed new CATT-S architecture, where convolutional layers extract local features and Transformer self-attention models dependencies across sequences of A-scans, reached 99.4% accuracy on experimental PAUT data with precision 0.914, recall 0.896, F1-score 0.905, and ROC–AUC 0.997; while more computationally demanding than CNN, CATT-S can still run in real time on CPU after ONNX conversion. Input sequence length was fixed to 50 signals, and to speed up complete scan processing, we implemented parallel chunk-based prediction, which keeps inference practical despite high CPU load.

Thus, we successfully improved the process of finding defects in the PAUT data using the standard SNR algorithm, although not for all possible data variations. Also, the developed new CATT-S architecture was optimized for parallel processing of several A-scan signal sequences at once using only the CPU, so it can be used for real-time defect detection in PAUT data.

Future work can be focused on improving this approach to apply it for more variation in PAUT welds and tests in real-time defect detection in PAUT.

## Figures and Tables

**Table 1:** Key variable parameters for simulated weld dataset collection.

Parameter Varied	Range/Values	Notes
Root face (RF)	0–3 mm	Variation in root face thickness
Gap (ζ)	1–4 mm	Joint gap between plates
Thickness (*t*)	5.6–18 mm	Variation in plate thickness
Angle (α)	70°, 75°	Weld V-groove angle
Defect types	Cracks, Porosity, Inclusions	Defects variation placed in weld root and weld toe
Non-defective samples	Included	Various geometrical profiles without flaws
Structural noise	Varied amplitude & density	Simulated inspection conditions
Dataset size	∼200 S-scan samples	Each S-scan consists of 39 A-scan signals
Augmentation	×2.5	Variations in noise maps and signal recombination

## Data Availability

The data supporting the findings of this study are not publicly available due to security restrictions of the providing company. However, the data may be made available upon reasonable request and subject to review.
